# Structural Insights into the Active Site Formation of DUSP22 in N-loop-containing Protein Tyrosine Phosphatases

**DOI:** 10.3390/ijms21207515

**Published:** 2020-10-12

**Authors:** Chih-Hsuan Lai, Co-Chih Chang, Huai-Chia Chuang, Tse-Hua Tan, Ping-Chiang Lyu

**Affiliations:** 1 Institute of Bioinformatics and Structural Biology, Department of Life Science, National Tsing Hua University, Hsinchu 30013, Taiwan; chsuan.l@gmail.com (C.-H.L.); cochihchang@gmail.com (C.-C.C.); 2 Immunology Research Center, National Health Research Institutes, Zhunan 35053, Taiwan; cinth@nhri.org.tw (H.-C.C.); ttan@nhri.org.tw (T.-H.T.)

**Keywords:** hydrogen bonding network, active site, N-loop, DUSP22, DUSPs, Cys-based PTPs

## Abstract

Cysteine-based protein tyrosine phosphatases (Cys-based PTPs) perform dephosphorylation to regulate signaling pathways in cellular responses. The hydrogen bonding network in their active site plays an important conformational role and supports the phosphatase activity. Nearly half of dual-specificity phosphatases (DUSPs) use three conserved residues, including aspartate in the D-loop, serine in the P-loop, and asparagine in the N-loop, to form the hydrogen bonding network, the D-, P-, N-triloop interaction (DPN–triloop interaction). In this study, DUSP22 is used to investigate the importance of the DPN–triloop interaction in active site formation. Alanine mutations and somatic mutations of the conserved residues, D57, S93, and N128 substantially decrease catalytic efficiency (*k*_cat_/*K*_M_) by more than 10^2^-fold. Structural studies by NMR and crystallography reveal that each residue can perturb the three loops and induce conformational changes, indicating that the hydrogen bonding network aligns the residues in the correct positions for substrate interaction and catalysis. Studying the DPN–triloop interaction reveals the mechanism maintaining phosphatase activity in N-loop-containing PTPs and provides a foundation for further investigation of active site formation in different members of this protein class.

## 1. Introduction

**Figure 1:**
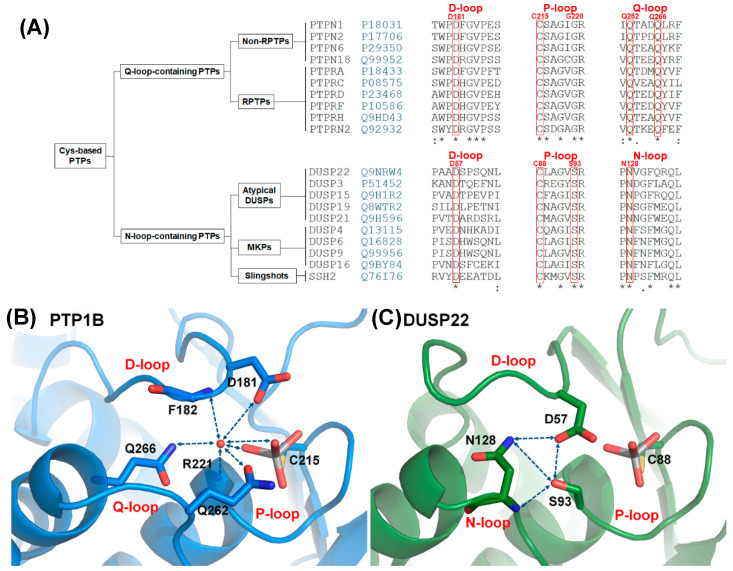
The D-, P-, N-triloop (DPN–triloop) interaction in N-loop-containing protein tyrosine phosphatases (PTPs). (**A**) The sequence alignment showed the difference between Q-loop-containing PTPs and N-loop-containing PTPs. N-loop-containing PTPs had an N-loop motif (PNXXF), which was different from the Q-loop motif (QXXXQ). The key residues (red) in PTP1B and DUSP22 are labeled above the sequence alignment. (**B**) In the structure of PTP1B (PDB ID: 3I80), structural water (red ball) can form the hydrogen bonding with the side chain of D181, the side chain of Q262, the side chain of Q266, the backbone amide of F182, the backbone amide of R221, and the oxygens of the ligand in active site. This water-mediated DPQ-triloop interaction connected the D-loop, the P-loop, the Q-loop, and the ligand, and aligned these residues to the correct position. (**C**) In the structure of DUSP22 (PDB ID: 6LVQ), the active site contained a hydrogen bonding network named the DPN–triloop interaction, which was formed by three residues: D57 in the D-loop, S93 in the P-loop, and N128 in the N-loop. The side chains of D57, S93, and N128 formed hydrogen bonds with each other, and the backbone amide of N128 formed hydrogen bonding with the side chain of S93. Hydrogen bonding is displayed as blue dotted lines, and the residues as well as the ligand, VO_4_, are shown by sticks.

Cysteine-based protein tyrosine phosphatases (Cys-based PTPs) are important enzymes that remove phosphate from phosphorylated residues and regulate several signaling pathways in cellular processes, including cell differentiation, proliferation, and immune response [1,2,3]. The somatic mutations on Cys-based PTPs can disrupt cellular processes and induce high risk of human diseases [4,5,6]. Cys-based PTPs consist of two major subfamilies: classical PTPs and dual-specificity phosphatases (DUSPs) [2]. The phosphatase domains in classical PTPs and DUSPs are formed by twisted β-sheets surrounded by α-helices. The active site is a crevice on the surface of the structure. The PTP signature motif, the P-loop (CX_5_R), is located at the bottom of this crevice, and it provides the binding site as well as a conserved cysteine to trigger catalytic activation [1,2]. The loops surrounding the P-loop play important roles in supporting catalytic reactions. The D-loop (WPD-loop) provides a conserved aspartate to act as a general acid/base during the catalytic reaction, and the Q-loop provides the conserved glutamines to stabilize water molecules for hydrolysis of the cysteinyl-phosphate intermediate and correct positioning of the active site residues [1,7,8,9]. The active site, which comprises the D-loop, P-loop, and Q-loop, is highly conserved in classical PTPs, and the mechanism is well understood. However, the Q-loop sequence varies in DUSPs [1]. Several members of DUSPs replace the Q-loop with an N-loop, which has a conserved sequence of PNXXF, in contrast to the Q-loop motif (QXXXQ) (Figure 1A) [10]. The first crystal structure of DUSPs is DUSP3, published by Saper’s group in 1996 [11], while the N-loop is described by Johnson’s group in 2012 [10]. The active site, which consists of the D-loop, P-loop, and N-loop, is present in 46% of DUSPs but has not been clearly investigated. These two groups of PTPs include more than half of the members in Cys-based PTPs; Q-loop-containing PTPs (classical PTPs) constitute 32% of the 116 members in Cys-based PTPs [2,12] while N-loop-containing PTPs constitute 25%. Since the two groups are clearly divided in phylogenetic relationships [13], investigation of N-loop-containing PTPs can provide information for further understanding the relationship between the structure and catalytic activity in the Cys-based PTP family.

PTP1B (PTPN1) is a well-known model for studying the reaction cycle in Q-loop-containing PTPs. The reaction cycle consists of two steps and three loops that participate in the reaction: the D-loop, P-loop, and Q-loop (Figure S1) [1,2]. In the first step, the P-loop provides arginine to stabilize substrate binding, and nucleophilic cysteine attacks the phosphorus atom. At this time, catalytic aspartate (D181) in the D-loop acts as a general acid to donate a proton for substrate dissociation and results in a cysteinyl-phosphate intermediate. In the second step, conserved glutamine (Q262) in the Q-loop motif rotates toward the ligand and acts as an anchor to stabilize the position of water for catalytic aspartate [7,8]. Catalytic aspartate in the second step acts as a general base that accepts protons from water and results in nucleophilic water, facilitating hydrolysis of the cysteinyl-phosphate intermediate and releasing phosphate [1,7]. The catalytic reaction is probably similar between Q-loop-containing PTPs and N-loop-containing PTPs because all of them have the P-loop and D-loop [1], but the different sequences in the Q-loop motif and N-loop motif suggest that they might have different roles in active site formation.

More differences between the two groups can be found in the active site and the selected ligands. The Q-loop-containing PTPs have a deep active site pocket with a depth of ~9 Å, and the substrate is phosphotyrosine (pTyr). The N-loop-containing PTPs have a shallow active site pocket of ~6 Å, and the substrates include pTyr as well as pThr/pSer [1,2,11,14]. In the Q-loop-containing PTPs, the active site contains a structural water molecule that interacts with a catalytic aspartate in the D-loop and the conserved glutamines in the Q-loop [7,9]. This structural water molecule (W1) is highly conserved in the crystal structures of Q-loop-containing PTPs [7], and interacts with the D-loop, P-loop, Q-loop, and ligand that align the residues in the active site (Figure 1B) [8,9]. In contrast, the catalytic aspartate in the D-loop is exposed on the surface of N-loop-containing PTPs and interacts with a serine in the P-loop and an asparagine in the N-loop through a hydrogen bonding network [10]. The interaction between the serine and aspartate in N-loop-containing PTPs replaces the position of structural water in Q-loop-containing PTPs [11], and the hydrogen bonds contributed by the serine and asparagine are described to be involved in maintaining the catalytic aspartate in a catalytically favorable conformation [10], but the mechanism has not been clarified. To investigate the role of this hydrogen bonding network, somatic mutations of serine and asparagine are searched in cancer database, and DUSP22 includes both S93 and N128 mutations in the P-loop and N-loop (Tables S1 and S2) [15,16].

DUSP22 is a small phosphatase containing 184 residues, and the sequence contains a catalytic domain as well as an N-terminal myristoylation site [17,18]. This enzyme is broadly expressed in different tissues and plays important roles in the regulation of signaling pathways, such as inactivating the T cell receptor (TCR) signaling pathway, activating the JNK signaling pathway, and regulating the phosphorylation of focal adhesion kinase (FAK) [17,19,20,21,22]. DUSP22-deficient animal models exhibit risks of autoimmunity, and downregulation of DUSP22 in patients exhibits a strong relationship with systemic lupus erythematosus (SLE) and inflammatory bowel disease (IBD) [19,23,24]. The phosphatase domain of DUSP22 consists of five twisted β-sheets and six α-helices, and the structural scaffold is similar to that of DUSP3 (VHR) [17], while DUSP22 does not contain an N-terminal α-helix surrounded by the N-loop. The P-loop is located between β5 and α3 and forms a shallow active site with a catalytic aspartate, D57, at a depth of 4.5 Å [17]. In this region, the catalytic activity is facilitated by C88 in the P-loop and D57 in the D-loop [17], and the hydrogen bonding network is formed by D57 in the D-loop, S93 in the P-loop, and N128 in the N-loop. We mutated these residues (D57, S93, and N128) to study whether the DPN–triloop interaction is associated with catalytic activity as well as structural formation and reveals a crucial role that is probably applicable to all N-loop-containing PTPs.

## 2. Results

### 2.1. Characteristics of the DPN–triloop Interaction

The hydrogen bonding network in N-loop-containing PTPs contains three conserved residues, namely, aspartate in the D-loop, serine in the P-loop, and asparagine in the N-loop (Figure 1A). The sequence alignment indicated that these residues were highly conserved in N-loop-containing PTPs, which included three families: atypical DUSPs, MKPs, and Slingshots (Figure 1A). The members included 26 phosphatases and 3 pseudophosphatases (DUSP27, STYX, and STYXL1) (Figure S2), suggesting that the DPN–triloop interaction not only played a role in catalysis but also participated in structural formation. In DUSP22, the hydrogen bonding network was formed by the side chain of D57 in the D-loop, S93 in the P-loop, and N128 in the N-loop. DUSP22 not only contains the hydrogen bonds which formed with the side chain of D57 but also includes the hydrogen bonds formed between S93 and N128 through the side chains and backbone amide (Figure 1C). This hydrogen bonding network was described as the DPN–triloop interaction in this study, and it probably contributed to stabilizing the three loops similar to the water-mediated DPQ-triloop interaction in Q-loop-containing PTPs (Figure 1B). To confirm that the DPN–triloop interaction was important for forming the structure of the active site, we mutated D57, S93, and N128 to detect phosphatase activity.

### 2.2. The Role of DPN–triloop Interaction in Phosphatase Activity

The residues, D57, S93, and N128, were mutated to study their effect on catalytic activity via a *p*-nitrophenyl phosphate (pNPP) kinetic assay. D57 is a catalytic aspartate [17], and the role of catalytic aspartate has been verified by alanine and asparagine mutations in previous studies [25,26]. S93 and N128 sites had unknown effects. We referred to the somatic mutated residues that were equivalent to S93 and N128 in different N-loop-containing PTPs in the cancer databases and found several reports, including S285L in DUSP4, S298G in DUSP6, S249F in DUSP16, S155F in DUSP19, S111N in DUSP21, N297S in DUSP2, N125D in DUSP15, N160D in STYX, N168K in DUPD1, and N453K in SSH3 (Table S1) [15,16]. To investigate the roles of these residues and the effects of somatic mutations, S93 and N128 were replaced with alanine and the referred mutants (Tables S1 and S2). N128D was selected due to similar side chain and enough protein yield, and S93N was the only S93 site mutant that induced enough protein yield.

The kinetic parameters of DUSP22 and the mutations in the DPN–triloop interaction are listed in Table 1. The enzyme kinetic of wild-type (WT) DUSP22 showed that the *K*_M_ was 0.64 mM and the *k*_cat_ was 0.65 s^−1^, which were similar to previous published results [27]. The mutations of D57 decreased *k*_cat_ by 26–31 times, while the *K*_M_ values were different between D57N and D57A. The *K*_M_ of D57N was similar to that of the WT, but the *K*_M_ of D57A was increased more than 8 times. The mutations of S93 and N128 increased *K*_M_ and decreased *k*_cat_ as well. S93A and S93N increased the *K*_M_ by 14–19 times, and the *k*_cat_ declined more than 10 times. N128A and N128D increased the *K*_M_ more than 30 times, but the *k*_cat_ values were different. N128D decreased the *k*_cat_ by 16 times, while the *k*_cat_ of N128A only decreased 2.5 times. The catalytic efficiency (*k*_cat_/*K*_M_) indicated that the mutations on D57, S93, and N128 decreased 40–500 times. These results reflected important information indicating that the mutations affected both substrate affinity and catalytic activity and were associated with disruption of the DPN–triloop interaction. The primary difference between D57N and the other mutations was the *K*_M_ value. The asparagine of D57N probably maintained the DPN–triloop interaction due to the similar side chain, but the other mutations did not maintain the interaction. The alanine mutations in D57, S93, and N128, might interfere with the formation of the DPN–triloop interaction, and S93N and N128D might disrupt the interaction through longer side chain and charge repulsion. Importantly, both mutations of S93 and N128 decreased the catalytic efficiency (*k*_cat_/*K*_M_) by more than 10^2^ times, revealing that S93 and N128 were necessary for maintaining phosphatase activity. The detailed structural studies of the DPN–triloop interaction were further investigated by NMR spectroscopy and protein crystallography.

### 2.3. D57, S93, and N128 Mutants Perturbed the Active Site Conformation in the Solution Structure

**Figure 2:**
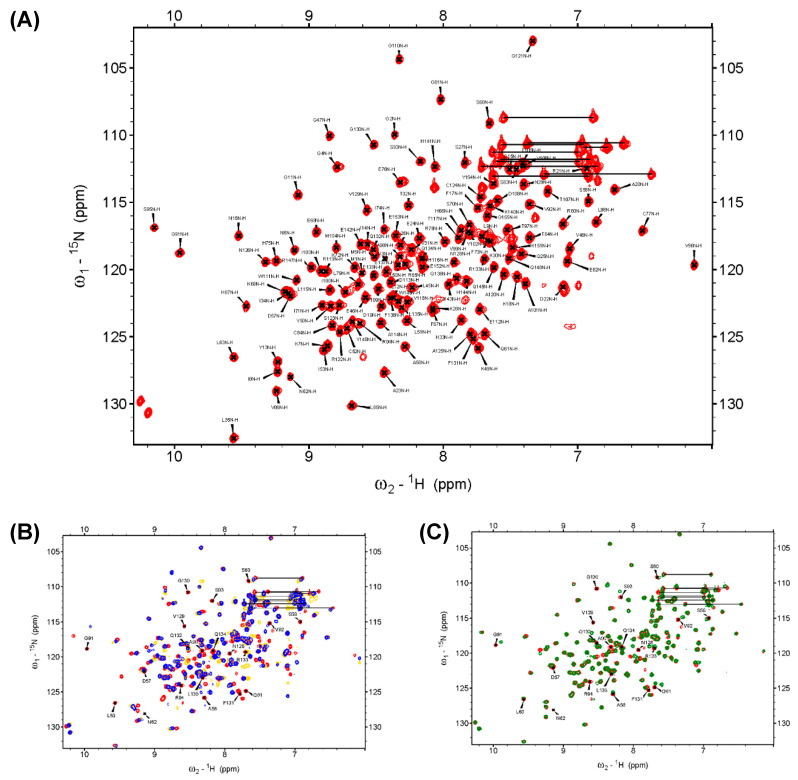
The backbone assignment of DUSP22_WT and the superimposed ^1^H-,^15^N-HSQC spectra of the WT, S93N, N128A, and D57N. (**A**) The backbone amide resonance of residues in the WT is assigned on the ^1^H-,^15^N-HSQC spectrum. It exhibited 139 backbone amide resonances of 150 expected resonances (5 prolines removed) in DUSP22 and showed 93% completion. (**B**) The ^1^H-,^15^N-HSQC spectrum of the WT (red) was superimposed with the spectra of S93N (yellow) and N128A (blue). The spectra indicated that the mutants critically perturbed the solution structures and shifted several residues. (**C**) The ^1^H-,^15^N-HSQC spectrum of the WT (red) was superimposed with the spectra of D57N (green), which revealed the region perturbed by the DPN–triloop interaction. The labeled amino acids on the spectra were the residues in the D-loop, P-loop, and N-loop of the WT.

**Figure 3:**
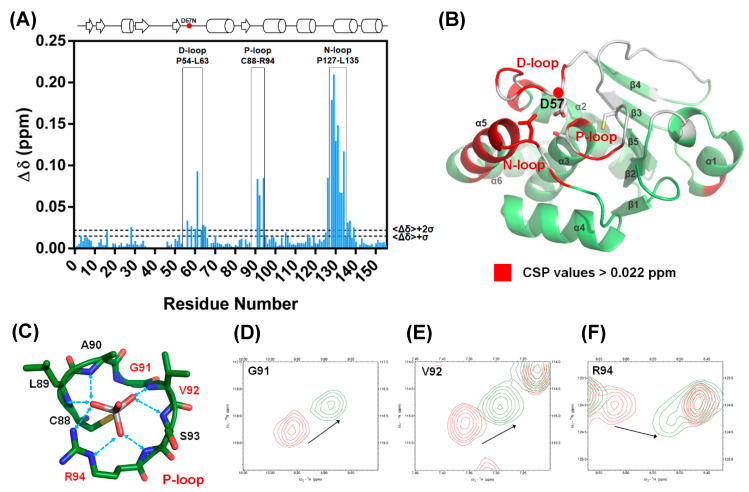
The D57N mutant exhibited a perturbed conformation as a result of DPN–triloop interactions. (**A**) The catalytic residue, D57, was replaced with asparagine, and the chemical shift perturbation (CSP) values were calculated. The threshold was set at Δδ + 2σ, and the value was 0.022 ppm. The data indicated that the residues in the D-loop, P-loop, and N-loop were specifically perturbed by the D57N mutant. (**B**) The residues with CSP values greater than Δδ + 2σ are labeled on the DUSP22 structure in red. These results revealed the region that was perturbed by DPN–triloop interaction, including the D-loop, P-loop, N-loop, and α-helix 5 (α5). The spaces and white residues were non-assigned residues in the backbone assignment, including proline and disappeared signals in the 3D spectra. (**C**–**F**) The P-loop is the binding site, and the residues in the P-loop share their backbone amides as well as side chains to interact with the ligands. The ^1^H-,^15^N-HSQC spectrum of the WT (red) was superimposed with the spectrum of D57N (green) to show that the P-loop residues, G91, V92, and R94, were perturbed by disruption of the DPN–triloop interaction.

NMR spectroscopy was used to identify the region associated with the DPN–triloop interaction in DUSP22. The backbone assignment of WT spectra was finished with 93% completion, and most of the residues in the D-loop, P-loop, and N-loop were detectable, including A56-S58, S60-L63, A90-R94, and N128-L135 (Figure 2A). The ^1^H-,^15^N-HSQC spectra of S93N and N128A were collected to observe the perturbation. The spectra indicated that these mutants nearly perturbed the entire phosphatase domain of DUSP22. The D57, S93, and N128 signals disappeared, and the surrounding residues were perturbed in the structures (Figure S3). Unfortunately, S93N and N128A were difficult to assign because they were unstable in the triple resonance experiments. We used D57N to observe how the DPN–triloop interaction perturbed the structure in the solution conformation. The D57N mutant was stable, and the residues located in the D-loop, P-loop, and N-loop could be identified. Comparison of the ^1^H-,^15^N-HSQC spectra of the WT and D57N showed that most backbone amide resonances in D57N were similar to those in the WT (Figure 2C), whereas the perturbed regions (more than ∆δ + 2σ) were primarily located on the D-loop, P-loop, N-loop, and α-helix 5 (α5) (Figure 3A,B). The D57N mutation not only obviously changed the D-loop itself but also significantly perturbed the residues in the P-loop and N-loop.

The result was a local perturbation, revealing that the three loops can interact and perturb each other in solution conformation. The P-loop is a critical binding structure that provides the backbone amide and the side chain of arginine to interact with the phosphate groups of substrates (Figure 3C) [1,11]. In the perturbed region, we observed that the P-loop residues, G91, V92, and R94, were shifted in the D57N spectra and disappeared in the S93N and N128A spectra (Figure 2B and Figure 3D–F). This revealed the conformational change in the P-loop, which probably affected the binding effect to reduce phosphatase activity. Moreover, the highest chemical shift perturbation (CSP) value was located on the N-loop motif, which perturbed the connected α-helix, α5, and induced high CSP values (Figure 3B), suggesting that the conformational changes in the D-loop, P-loop, and N-loop can affect the surrounding structure. In this light, the perturbations in S93N and N128A mutants probably resulted from the conformational changes due to the disruption of the DPN–triloop interaction, indicating that more residues were perturbed by the mutations in solution structure (Figure 2B and Figure S3). These results revealed that D57, S93, and N128 contributed to forming the conformation of each loop and stabilized the structure of the active site.

### 2.4. The Structural Role of the DPN–triloop Interaction in Crystal Structures

The crystal structures of C88S, C88S/S93A, C88S/S93N, WT_VO_4_, N128A, and N128D were determined in this study. All of the structures contained a ligand, PO_4_, VO_4_, or SO_4_, bound to the P-loop in the active site. The structures of WT_VO_4_ and C88S_PO_4_ were similar, and their ligands were different from the previously published structures (PDB ID: 1WRM and 4WOH), which bound pTyr-like ligands [17,28]. The binding of the pTyr-like ligand was representative of the first step in the catalytic reaction, and the binding of vanadate represented the second step (Figure S1) [7]. The two structures were similar. Unlike Q262 in PTP1B, N128 did not rotate to stabilize water during the two catalytic steps (Figure S4). The DPN–triloop interaction was maintained in the catalytic reaction, suggesting that the stable interaction among the three loops was necessary for catalysis.

**Figure 4:**
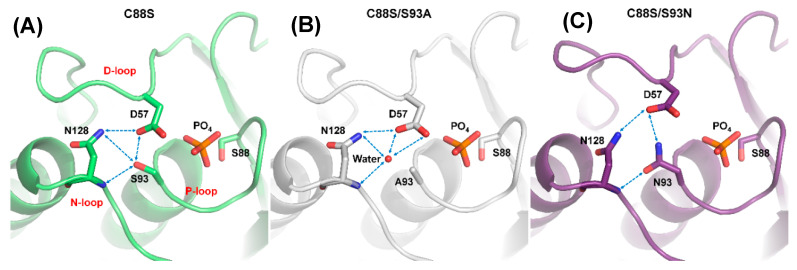
Disruption of DPN–triloop interaction in the crystal structures of S93 mutants. (**A**–**C**) The crystal structures of C88S (green), C88S/S93A (white), and C88S/S93N (purple) indicated how the mutation affected the hydrogen bonding network. The hydrogen bonds are shown by blue dotted lines, and a water molecule is shown as a red ball. (**A**) The crystal structure of C88S was prepared to compare with the S93 mutants, and the DPN–triloop interaction was unchanged. (**B**) S93 was replaced with alanine, and the structure was similar to that of C88S. A water molecule (W61) participated in forming the hydrogen bonding with the side chain of D57, the side chain of N128, and the backbone amide of N128. (**C**) S93 was replaced with a somatic mutant, asparagine. N93 formed the hydrogen bonding with the side chain of D57 and the backbone amide of N128.

The S93 mutants were combined with a second mutation, C88S, to produce proteins for growing better crystals, which improved the resolution (<1.6 Å) and helped to determine the structures. The crystal structure of C88S was prepared to compare with the double mutants, and it formed the DPN–triloop interaction, which was similar to the WT (Figure 4A). In the crystal structure of C88S/S93A, the overall structure was similar to that of C88S, and the side chains of D57 and N128 also formed a hydrogen bond (Figure 4B). A water-mediated hydrogen bonding network was formed among the side chains of D57 and N128, as well as the backbone amide of N128. The position of this water molecule was above the original position of S93 and it did not form a hydrogen bond with the P-loop. A93 in the structure was independent of the water-mediated hydrogen bonding network, and the comparison of B-factor between C88S and C88S/S93A showed that the positional disorder increased on the three loops (Figure S5). The S93A mutation probably resulted in the unstable active site structure and decreased the catalytic efficiency. In contrast, the N93 in the crystal structure of C88S/S93N had a different effect and formed the hydrogen bonding with the side chain of D57 and the backbone amide of N128 (Figure 4C). The side chain of N128 also interacted with the side chain of D57. The DPN–triloop interaction formed but was not precise. This DPN–triloop interaction lacked a hydrogen bond between the side chains of N93 and N128, and the positions of D57 and N128 were shifted due to a spatial barrier from N93. The decrease in catalytic efficiency in S93N was associated with this conformational change. This result indicated that the formation of the DPN–triloop interaction through S93 induced precise spatial localization to stabilize active site structure and maintain phosphatase activity.

**Figure 5:**
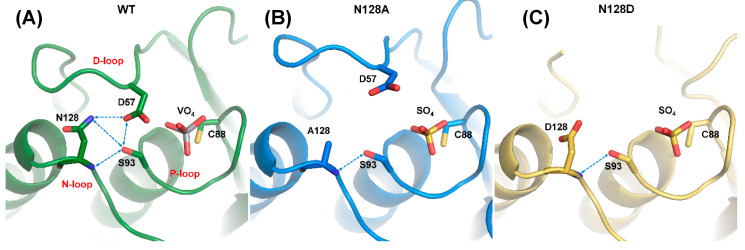
Disruption of DPN–triloop interaction in the crystal structures of N128 mutants. (**A**–**C**) The crystal structures of WT (green), N128A (blue), and N128D (yellow) indicated how the mutation affected the hydrogen bonding network and D-loop. The hydrogen bonds are shown by blue dotted lines. (**A**) The crystal structure of WT was co-crystallized with vanadate, and the DPN–triloop interaction was maintained. (**B**) N128 was replaced with alanine, and the conformation of the D-loop changed. Only one hydrogen bond was formed between the side chain of S93 and backbone amide of N128. (**C**) N128 was replaced with a somatic mutant, aspartate. The D128 induced charge repulsion with D57 and resulted in a dynamic D-loop that had unclear electron density.

Structural alignment among N128A, N128D, and WT_VO_4_ revealed that N128 participated in forming the conformation of the D-loop (Figure 5). In the N128A structure, one hydrogen bond was formed between the side chain of S93 and the backbone amide of A128. The interaction between D57 and N128 was broken, and no water-mediated hydrogen bonds connected them (Figure 5B). The D-loop was shifted away in the N128A mutant, indicating that the hydrogen bond from S93 was not sufficient to stabilize the D-loop. A similar result was observed in the N128D structure. The negative charge from D128 in the N128D mutant induced charge repulsion against D57, which resulted in unclear electron density in the conformation of the D-loop and slightly shifted the N-loop (Figure 5C). These results clearly indicated that the N128-D57 interaction anchored the D-loop and stabilized the conformation of the active site.

## 3. Discussion

N-loop-containing PTPs are the second largest family among Cys-based PTPs, and the characteristic of this family is formation of the DPN–triloop interaction in the active site. This conformation is crucial to maintain the phosphatase activity in DUSP22, and the forming mechanism is different from the water-mediated DPQ-triloop interaction in Q-loop-containing PTPs (Figure 1).

The structural difference between DPN–triloop interaction and DPQ-triloop interaction is probably associated with the specificity of the selection of ligands. These two groups have a common ancestral gene and divided during evolution [13]. N-loop-containing PTPs have a shallow and concise active site structure to interact with pSer, pThr, and pTyr, while Q-loop-containing PTPs extend the space of the active site pocket to specifically select pTyr. The purpose of the hydrogen bonding network in active sites is similar between DPN–triloop interaction and DPQ-triloop interaction. It positions the active site residues at a precise site for substrate interaction and catalysis. Our experiments provide evidence showing that completely forming the DPN–triloop interaction contributes to stabilizing the conformation of the active site.

The D-loop and P-loop need to be stabilized in the active site during the catalytic reaction. The DPN–triloop interaction stabilizes them and makes the three loops become a configuration. This means that the conformational change on each loop can perturb the others and change the conformation of the active site. Perturbation of the backbone amide in the P-loop is observed in the solution structure by disrupting the DPN–triloop interaction (Figure 2 and Figure 3), which probably interferes with ligand binding as well as catalysis, and the perturbed conformation in S93A and S93N decreases the catalytic efficiency by 150–260 times (Table 1). In the crystal structure, the S93A mutant shows that the P-loop is independent from the interaction of the hydrogen bonding network and the B-factor is increase in the three loops (Figure 4B and Figure S5), suggesting that the conformation of the D-loop, P-loop, and N-loop cannot be stabilized. Even though S93N interacts with N128 and D57 in the crystal structure, the positions of D57 and N128 are different from those in the WT (Figure 4C), and the N-loop and the D-loop are shifted. These results reveal that the conformation of the three loops is affected by the DPN–triloop interaction, and S93 contributes to the precise spatial position for forming hydrogen bonds. The larger side chains, asparagine, leucine, and phenylalanine (Table S1), are not suitable for this position, and these somatic mutations may interfere with the position of active site residues and abolish the phosphatase activity. Similar results are observed in N128 mutants. Disruption of the DPN–triloop interaction by N128A and N128D critically decrease the catalytic efficiency by 100–500 times (Table 1) because it induces conformation changes in the D-loop (Figure 5B–C) and perturbs the conformation of the P-loop (Figure 2B and Figure S3). The conformational change of the active site is the main reason for *K*_M_ increase, which is observed in most mutations. The *k*_cat_ is associated with the interaction between D57 and S93, which can explain the difference between N128A and N128D. In the N128D structure, the charge repulsion induces D57 to be unable to interact with S93 and substrate, which decreases the *k*_cat_. N128A does not form a barrier against D57, so D57 can interact with S93 due to D-loop dynamics and catalyze the substrate, while this interaction is not stable and induces a high *K*_M_. Consistently, the decrease in *k*_cat_ is observed in the S93A mutant because D57 cannot form a stable interaction with S93. In the S93N mutant, N93 is a barrier that blocks D57 close to the substrate and decreases *k*_cat_. The results of S93 and N128 verify that forming hydrogen bonds among D57, S93, and N128 are necessary to maintain the phosphatase activity. The DPN–triloop interaction stabilizes the residues in three loops at precise position and form the active site for facilitating the catalysis.

The residues in the region of the DPN–triloop interaction are potential sites for phosphatase activity regulation. Several somatic mutations are found in this region (Tables S1 and S2). Some of the mutations are on the critical residues, such as D57, C88, and R94, which directly abolish catalytic activity and disrupt substrate binding [29]. The mutation of S93F, deletion of N128, and equal mutations in other N-loop-containing PTPs (Table S1) [15,16] can decrease the catalytic efficiency by disruption of the DPN–triloop interaction, and the mechanism is described in this study. Similarly, the mutations surrounded by D57, S93, and N128 might regulate phosphatase activity by changing the conformations of the D-loop, P-loop, and N-loop. In previous study, the allosteric sites in DUSP3 (VHR) and DUSP10 (MKP5) are reported, and these allosteric sites are surrounded by the N-loop and can affect the phosphatase activity [30,31]. Whether the mutations shown in Tables S1 and S2 participate in the process of cancer development is worth studying because N-loop-containing PTPs regulate several signaling pathways, and the deficiency or downregulation of these PTPs is frequently observed in cancer development [32,33]. Drug development to stabilize the DPN–triloop interaction is a possible strategy to benefit cancer treatment. However, some of the members participate in the promotion of cancer development, such as STYX, SSH1, and SSH3 [34,35,36]. Drug development requires more structural information for support, and our studies may serve as a foundation for further investigation of treatments focusing on N-loop-containing PTPs.

## 4. Materials and Methods

### 4.1. Protein Expression and Purification

The truncated DUSP22 (1–155 a.a.) was used in our experiments, and the purification method was modified from a previous study [17]. In brief, the truncated DUSP22 gene was ligated into pET21b (Merck KGaA, Darmstadt, Germany) with a hexa-histidine tag followed by a PreScission protease cleavage site (LEVLFQ/GP) on the N-terminus. Site-directed mutagenesis was performed by means of a KOD-plus Kit (Toyobo, Osaka, Japan). WT enzyme and most mutants were expressed by Rosetta 2 (DE3) (EMD Millipore, Billerica, MA, USA); meanwhile, the S93A mutant was expressed by BL21 (DE3) in a chaperone expression system (GroES and GroEL) (Takara Bio, Otsu, Shiga, Japan). The cells were grown at 37 °C for 3 h, and 1 mM IPTG was added to induce protein expression at 25 °C for 6–8 h or 16–20 °C overnight. Cell pellets were resuspended in buffer A (20 mM PIPES (pH 7.0), 100 mM NaCl) with 1 mM imidazole and then disrupted by sonication (Sonics & Materials, Newtown, CT, USA). The supernatant was loaded onto a TALON metal affinity resin (Takara Bio) and eluted with buffer B (20 mM PIPES (pH 7.0), 100 mM NaCl, 150 mM imidazole). The proteins were incubated with PreScission protease at 4 °C until complete cleavage of the His-tag, and loaded onto a size exclusion column (Sephacryl S-100 16/60) (GE Healthcare, Chicago, IL, USA) for elution by buffer A. The fractions were concentrated and stored at −20 °C until use.

### 4.2. Kinetic Assay

Phosphatase activity was detected by the cleavage of *p*-nitrophenyl phosphate (pNPP), and the method was modified from previous studies [26,37,38]. The kinetic assay was performed in reaction buffer (0.1 M sodium acetate, 0.05 M Tris, and 0.05 M Bis-Tris, pH 6.0). To calculate the *K*_M_ and *k*_cat_ values, the protein concentration and substrate concentration were adjusted. WT and N128A were performed at a final concentration of 0.1 μM, and D57A, D57N, S93A, S93N, and N128D were performed at a final concentration of 1 μM. pNPP substrate was prepared for 11–12 concentrations in different ranges (0.1–20 mM, or 1–50 mM with or without 80 mM). Proteins and substrate were mixed in equal ratios and incubated at 30 °C for 15 min. The reaction was stopped by adding 2 N NaOH, and the absorbance at 405 nm was measured by NanoPhotometer Pearl (Implen, Westlake Village, CA, USA). The results were collected in triplicate experiments and fitted to the Michaelis-Menten equation in GraphPad Prism version 8 (GraphPad Software, San Diego, CA, USA).

### 4.3. NMR Spectroscopy

NMR spectra were collected on Bruker Avance II 600 MHz and Bruker Avance III 850 MHz spectrometers (Bruker BioSpin, Rheinstetten, Germany) at 298 K. For backbone assignment, ^15^N-,^13^C-labeled DUSP22_155WT was prepared at a final concentration of 1 mM in NMR buffer (40 mM PIPES (pH 7.0), 100 mM NaCl, 10% D_2_O (*v/v*) and 0.16 mM DSS). The collected spectra included HSQC, HNCA, HN(CO)CA, HNCO, HNCACB, and HN(CO)CACB. The assignment of D57N was related to the WT spectra and confirmed by backbone assignment of the Cα spectra (HNCA, HN(CO)CA) as well as ligand titration. For comparison of the mutants, ^15^N-labeled proteins were prepared at the concentration of 0.1–0.2 mM to collect ^1^H,^15^N-heteronuclear single quantum coherence (HSQC) spectra. All spectra were processed by NMRpipe [39] and analyzed by Sparky [40]. The CSP values were calculated by the following formula:

$$CSP\;\left(ppm\right)=\sqrt{\frac{1}{2}\left(\delta_{HN}^{2}+\frac{\delta_{N}^{2}}{25}\right)}$$

The threshold of CSPs was set at two standard deviations above the mean (∆δ + 2σ), which was calculated from 10% trimmed data [30,41], and the residues with the CSP values above the threshold were labeled on the structure.

### 4.4. Protein Crystallization

Crystallization trials of WT_VO_4_, C88S, C88S/S93A, C88S/S93N, N128A, and N128D were performed by hanging drop vapor diffusion. WT, C88S, N128A, and N128D were prepared in buffer A while C88S/S93A and C88S/S93N were prepared in buffer A with 2 mM Li_2_SO_4_ to prevent aggregation. The details of the protein concentrations and the reservoir solution are listed in Table S3. Proteins were mixed with reservoir solution at an equal ratio (1:1) and incubated at 20 °C. The WT crystal grew in 24 h, and the crystal was soaked with a high concentration of VO_4_ in soaking solution (Table S3) for 48 h. The crystals of C88S, C88S/S93A, and C88S/S93N grew in 16–24 h, and those of N128A and N128D grew in 48 h.

### 4.5. X-Ray Data Collection and Structure Determination

The crystals were picked up and flash frozen in liquid nitrogen with 10–20% glycerol. X-ray data were collected by TLS_15A, TLS_13B, and TPS_05A at NSRRC (Hsinchu, Taiwan). The collected data were processed by HKL2000 [42]. Crystal structures were obtained from Phaser-MR in Phenix [43], and the search model was WT DUSP22 (PDBID: 1WRM). The ligands, VO_4_, PO_4_, and SO_4_, were built and fit into crystal structures by eLBOW and LigandFit in Phenix. Refinement was performed and built manually in Phenix and Coot [44]. The data collection and refinement statistics are listed in Table S4. The protein structures of DUSP22_WT and the mutants were generated with PyMOL [45].

### 4.6. Data Availability

The backbone assignment of DUSP22_WT has been deposited in the Biological Magnetic Resonance Data Bank (BMRB ID: 50156). The atomic coordinates and structure factors of WT_VO_4_, C88S, C88S/S93A, C88S/S93N, N128D, and N128A have been deposited in the Protein Data Bank (PDB ID codes 6LVQ, 6L1S, 6LMY, 6LOU, 6LOT, and 7C8S).

## 5. Conclusions

This study demonstrates the relationship between the DPN–triloop interaction and phosphatase activity in DUSP22. The DPN–triloop interaction forms the structure of the active site, and the mechanism differs from that of DPQ-triloop interaction. The DPN–triloop interaction stabilizes active site formation for substrate interaction and aligns the residues for catalysis. The high conservation of the D57 residue in the D-loop, the S93 residue in the P-loop, and the N128 residue in the N-loop explains the importance of the DPN–triloop interaction, which probably results in the active site formation in all N-loop-containing PTPs. Therefore, the DPN–triloop interaction may become a key for drug development to regulate the phosphatase activity of N-loop-containing PTPs. Our work provides a foundation for further study of active sites in different N-loop-containing PTPs and crucial information for investigating cancer mutations in the region of the DPN–triloop interaction.

## Figures and Tables

**Table 1:** Kinetic parameters of the mutations in the DPN–triloop interaction.

	*K*_M_ (mM)	*k*_cat_ (s^−1^)	*k*_cat_/*K*_M_ (s^−1^·M^−1^)
**WT**	0.64 ± 0.03	0.65 ± 0.01	1.0 × 10^3^
**D57A**	5.6 ± 0.7	0.025 ± 0.001	4.5
**D57N**	0.83 ± 0.07	0.021 ± 0.0005	25
**S93A**	12 ± 2	0.045 ± 0.003	3.8
**S93N**	8.8 ± 0.5	0.057 ± 0.001	6.5
**N128A**	26 ± 3	0.26 ± 0.01	10
**N128D**	21 ± 2	0.041 ± 0.002	2.0

## Supplementary Materials

Supplementary materials can be found at https://www.mdpi.com/1422-0067/21/20/7515/s1.

## Abbreviations

Cys-based PTPs	Cysteine-based protein tyrosine phosphatases
DUSPs	Dual-specificity phosphatases
NMR	Nuclear magnetic resonance
P-loop	Phosphate binding loop
