# Supplementary material for: Structural Insights into the Active Site Formation of DUSP22 in N-loop-containing Protein Tyrosine Phosphatases
Source: Int J Mol Sci. 2020 Oct 12;21(20):7515. doi: 10.3390/ijms21207515 (PMC7589817; doi:10.3390/ijms21207515)
Supplement: Supplementary file 1 [file ijms-21-07515-s001.pdf]

International Journal of *Molecular Sciences*

MDPI

Supporting Information

Structural Insights into the Active Site Formation of DUSP22 in N-loop-containing Protein Tyrosine Phosphatases

Chih-Hsuan Lai[1], Co-Chih Chang[1], Huai-Chia Chuang[2], Tse-Hua Tan[2], and Ping-Chiang Lyu[1*]

[1]Institute of Bioinformatics and Structural Biology, Department of Life Science, National Tsing Hua University, Hsinchu 30013, Taiwan; chsuan.l@gmail.com (C.-H.L.); cochihchang@gmail.com (C.-C.C.)

[2]Immunology Research Center, National Health Research Institutes, Zhunan 35053, Taiwan; cinth@nhri.org.tw (H.-C.C.); ttan@nhri.org.tw (T.-H.T.)

*Corresponding Author: Ping-Chiang Lyu;

E-mail: pclyu@mx.nthu.edu.tw; Tel.: 886-3574-2762; Fax: 886-3571-5934.

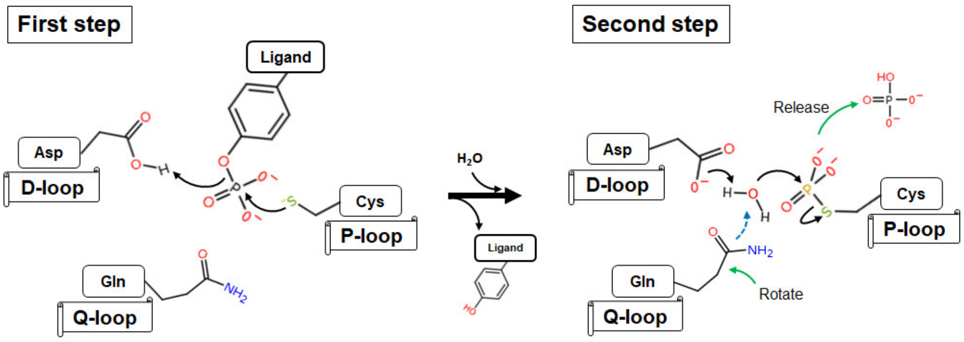

Figure S1. The catalytic reaction in Q-loop-containing PTPs. The model of two catalytic steps in PTP1B.

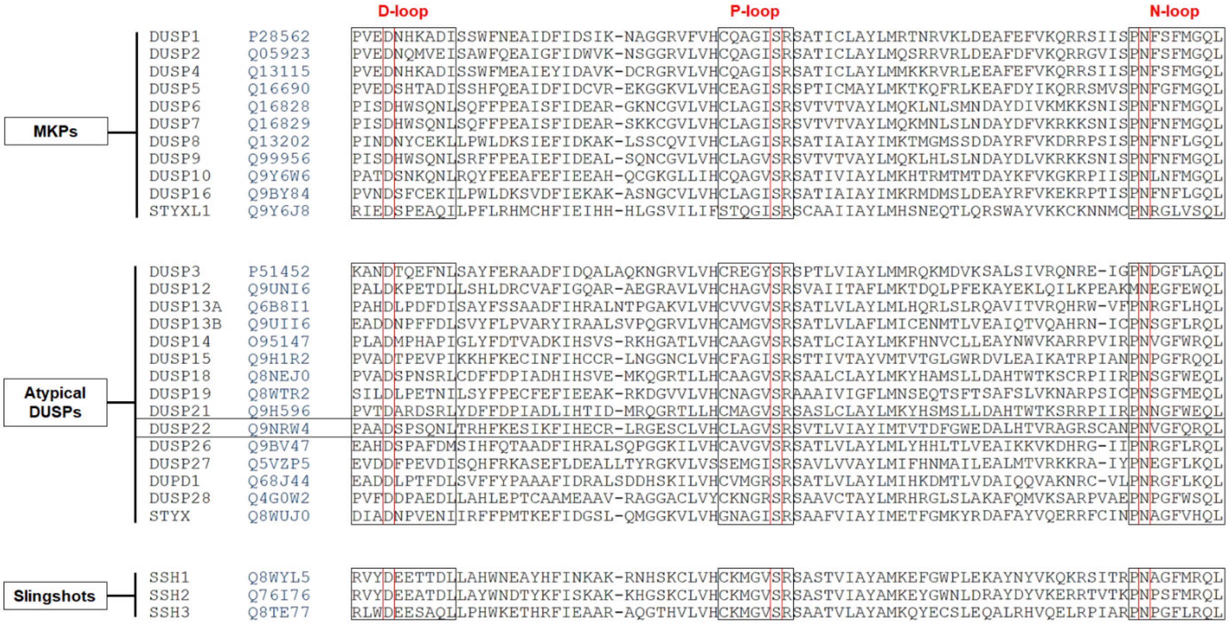

Figure S2. List of N-loop-containing PTPs. The 29 Cys-based PTPs contain the N-loop among three subfamilies, MKPs, atypical DUSPs, and Slingshots. All of the members contain the characteristic of the PND-triloop interaction (red circle), including the conserved aspartate in the D-loop, the conserved serine in the P-loop, and the conserved asparagine in the N-loop. The members include three pseudophosphatases (DUSP27, STYX, and STYXL1), which are inactive phosphatases because the catalytic cysteine is changed. DUSP11 and DUSP23 are not included in this alignment because they do not have this characteristic.

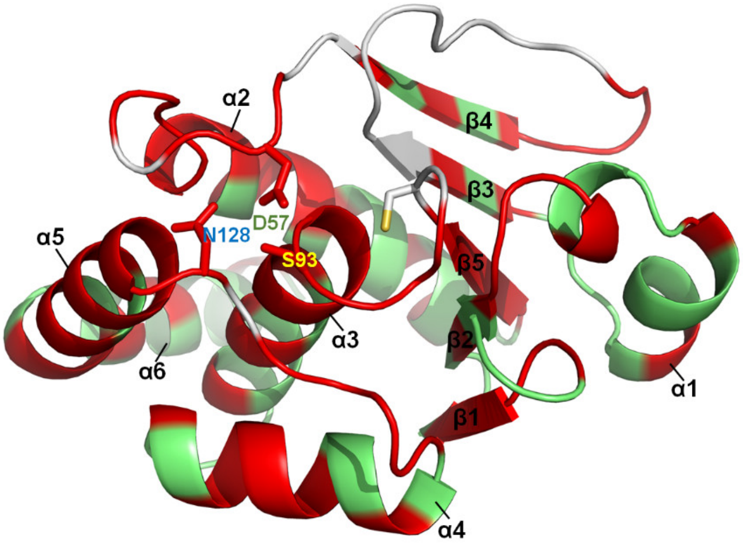

Figure S3. The perturbation region of S93N and N128A mutations. The chemical shifts of the mutations that differed from the WT are labeled on the protein structure in red. This includes the signal that probably disappeared in S93N and N128A mutations. S93N and N128A had similar effects in perturbing the solution conformation of the D-loop, P-loop, and N-loop. The residues in the surrounding structure were also affected by the conformational changes of three loops, such as β1, β5, and *α*2-*α*5. The white residues were nonassigned residues in the backbone assignment, including proline and the disappeared signals in the 3D spectra of WT.

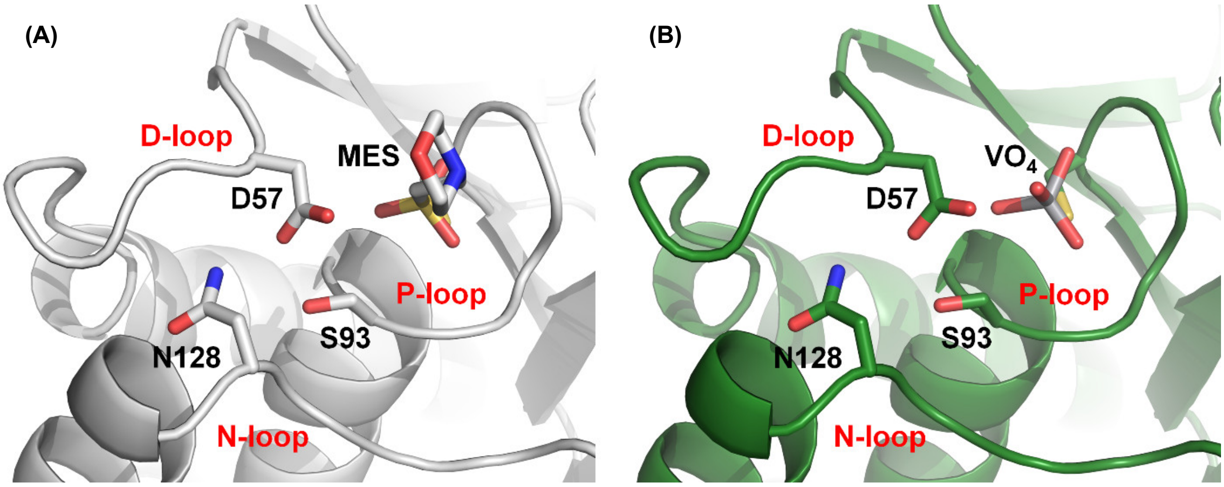

Figure S4. The two catalytic steps of DUSP22. (A) The crystal structure in the first catalytic step bound with a pTyr-like ligand, MES (PDB ID: 1WRM). (B) The crystal structure in the second catalytic step bound with a ligand, vanadate (VO_4). In alignment, N128 in DUSP22 was considered to replace the role of Q262 in PTPB. However, N128 in DUSP22 did not rotate during two catalytic steps as Q262 did in PTP1B. The N128 in the N-loop was far from ligand (>7 Å) and did not participate in water stabilization for hydrolysis.

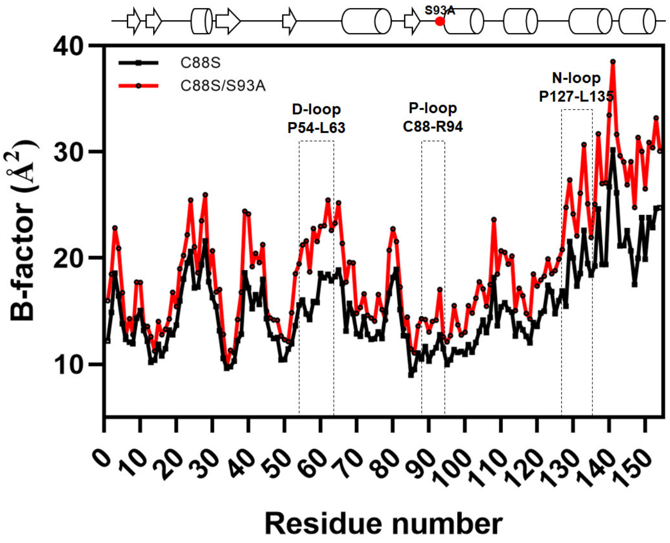

Figure S5. The B-factor of Cα in the crystal structure of C88S and C88S/S93A. The crystals of C88S and C88S/S93A grew in the same reservoir solution and formed the same space group, and so the comparison of B-factor reflected the positional disorder in the S93A mutant. C88S is shown by the black line, and C88S/S93A is shown by the red line. The S93A mutation induced the B-factor increase in the crystal structure, suggesting that the three loops became flexible and induced unstable active site.

Table S1. The somatic mutations equivalent to S93 (black) and N128 (red) in cancer databases

	Mutation	Tumor type	Tumor subtype	Reference ID[a]
**DUSP2**	**N297S**	Esophageal cancer	Esophageal adenocarcinoma	ICGC (ID: MU132377584)
**DUSP4**	**S285L**	Bladder cancer	Transitional cell carcinoma	COSMIC (ID: COSV53544999)
		Bladder cancer	Urothelial carcinoma	ICGC (ID: MU4624050)
		Colorectal cancer	Adenocarcinoma	
		Ovarian cancer	Serous cystadenocarcinoma	
**DUSP6**	**S298G**	Prostate cancer		ICGC (ID: MU121368750)
**DUSP15**	**N125D**	Lung cancer	Squamous cell carcinoma	ICGC (ID: MU1328471)
**DUSP16**	**S249F**	Liver cancer	Hepatocellular carcinoma	ICGC (ID: MU112731400)
**DUSP19**	**S155F**		Malignant melanoma	COSMIC (ID: COSV61193521)
		Breast cancer	ER-PR-positive carcinoma	COSMIC (ID: COSV61193521)
**DUSP21**	**S111N**	Colorectal cancer cell line	Adenocarcinoma	COSMIC (ID: COSV59133864)
**DUSP22**	**S93F**	Skin cancer	Basal cell carcinoma	COSMIC (ID: COSV60529532)
	**N128$_{deletion}$**	Colorectal cancer cell line	Adenocarcinoma	COSMIC (ID: COSV60529373)
**STYX**	**N160D**	Breast cancer	Ductal carcinoma	ICGC (ID: MU65717225)
**DUPD1**	**N186K**	Gastric cancer	Multiple histological subtypes	ICGC (ID: MU121096102)
**SSH3**	**N453K**	Bladder cancer	Invasive urothelial bladder cancer	ICGC (ID: MU131862676)

a. The somatic mutations are reported by the ICGC and COSMIC databases [1,2].

Table S2. The somatic mutations in the P-loop, D-loop, and N-loop of DUP22 in cancer databases

Loop	Mutation	Tumor type	Tumor subtype	Reference ID[a]
D-loop	A56V	Gastric cancer	Adenocarcinoma	ICGC (ID: MU1821692)
		Endometrial cancer	Uterine corpus endometrial carcinoma	
	D57N	Pediatric Brain Tumor	Multiple subtypes	ICGC (ID: MU127238269)
		Brain cancer	Glioblastoma multiforme	
	S58L	Skin cancer	Squamous cell carcinoma	COSMIC (ID: COSV60523881)
		Skin cancer	Cutaneous melanoma	ICGC (ID: MU4512779)
	P59S	Brain cancer	Lower grade glioma	ICGC (ID: MU130895128)
	P59Q	Lung cancer	Adenocarcinoma	ICGC (ID: MU131619769)
	L63M	Endometrial cancer	Uterine corpus endometrial carcinoma	ICGC (ID: MU129908241)
P-loop	C88Y	Brain cancer	Glioblastoma multiforme	ICGC (ID: MU129123398)
	A90V	Skin cancer	Cutaneous melanoma	ICGC (ID: MU129925934)
	G91A	Breast cancer	Ductal & lobular	ICGC (ID: MU131715533)
	G91W	Lung cancer	Adenocarcinoma	ICGC (ID: MU131760231)
	G91E	Skin cancer	Cutaneous melanoma	ICGC (ID: MU129904012)
	S93F	Skin cancer	Basal cell carcinoma	COSMIC (ID: COSV60529532)
	R94M	Lung cancer	Adenocarcinoma	ICGC (ID: MU131780295)
N-loop	$N128_{deletion}$	Colorectal cancer cell line	Adenocarcinoma	COSMIC (ID: COSV60529373)
	V129M	Gastric cancer	Adenocarcinoma	ICGC (ID: MU6171254)
	G130C	Esophageal cancer	Esophageal adenocarcinoma	ICGC (ID: MU37138925)
	G130D	Lung cancer	Adenocarcinoma	ICGC (ID: MU131506908)

a. The somatic mutations are reported by the ICGC and COSMIC databases [1,2].

Table S3. The crystallization conditions for the WT and mutants

Mutant	Protein concentration	Reservoir solution
**WT_VO_4**	6.1 mg/ml	0.1 M MES (pH 6.5), 2 mM $MnCl_2$, 0.1 mM Na_3VO_4, 25% PEG 4,000<br>Soaking solution: 0.1 M MES (pH 6.5), 5 mM Na_3VO_4, 25% PEG 4,000
**C88S**	12.7 mg/ml	0.2 M imidazole (pH 8.0), 0.4 M NaH_2PO_4, 1.6 M K_2HPO_4, 0.2 M NaCl
**C88S/S93N**	7.7 mg/ml	0.05 M arginine, 0.05 M glutamic acid (pH 7.5), 0.4 M NaH_2PO_4, 1.6 M K_2HPO_4, 0.2 M NaCl
**C88S/S93A**	3.5 mg/ml	0.2 M imidazole (pH 8.0), 0.4 M NaH_2PO_4, 1.6 M K_2HPO_4, 0.2 M NaCl
**N128D**	8.4 mg/ml	0.1 M PIPES (pH 6.7), 30% PEG 3,350, 0.2 M Li_2SO_4
**N128A**	7.0 mg/ml	0.1 M HEPES (pH 7.5), 30% PEG 3,350, 0.2 M Li_2SO_4

Table S4. Data collection and structural refinement statistics

	**WT_VO$_4$**	**C88S**	**C88S/S93N**	**C88S/S93A**	**N128D**	**N128A**
**Data collection**						
Space group	P 1 2 $_1$ 1	C 1 2 1	C 1 2 1	C 1 2 1	C 1 2 1	C 1 2 1
Cell dimensions						
a, b, c (Å)	36.1, 49.7, 39.8	89.6, 49.7, 39.8	90.0, 50.2, 40.9	89.7, 50.2, 39.7	90.3, 50.7, 41.1	89.8, 50.4, 40.2
α, β, γ (°)	90.0, 108.1, 90.0	90.0, 94.7, 90.0	90.0, 93.6, 90.0	90.0, 94.9, 90.0	90.0, 91.3, 90.0	90.0, 96.0, 90.0
Resolution (Å)	50.00-1.38 (1.43-1.38)	30.00-1.36 (1.41-1.36)	30.00-1.53 (1.58-1.53)	30.00-1.50 (1.55-1.50)	30.00-1.69 (1.75-1.69)	30.00 - 1.31 (1.36-1.31)
$R_{p.i.m.}$	0.048 (0.353)	0.020 (0.134)	0.023 (0.187)	0.034 (0.393)	0.028 (0.222)	0.033 (0.388)
I / sigma	18.9 (2.9)	36.2 (5.9)	46.5 (4.1)	22.5 (2.4)	24.4 (4.2)	22.0 (2.1)
Completeness (%)	97.9 (95.1)	97.3 (95.9)	98.3 (91.0)	99.5 (99.7)	98.4 (92.4)	98.6 (97.4)
Redundancy	5.6 (3.3)	3.8 (3.8)	3.4 (2.5)	3.6 (3.4)	3.5 (3.3)	3.7 (3.7)
**Refinement**						
Resolution (Å)	28.26-1.38 (1.43-1.38)	22.33-1.36 (1.41-1.36)	22.47-1.53 (1.58-1.53)	25.61-1.50 (1.55-1.50)	25.87-1.69 (1.75-1.69)	22.53 - 1.31 (1.36-1.31)
No. of reflections	27061	36406	27122	28187	20584	42475
R_{work} / R_{free}	0.2041/0.2287	0.1797/0.1954	0.1717/0.1957	0.1820/0.2129	0.1919/0.2192	0.1874 / 0.1965
No. of atoms						
Protein	1248	1248	1246	1243	1211	1241
Ligand/ion	5	5	5	5	5	5
Water	98	185	133	111	78	154
B-factors						
Protein	19.6	17.6	18.7	21.7	25.0	18.7
Ligand/ion	16.5	11.8	12.0	15.6	19.4	13.4
Water	28.4	29.7	29.6	32.5	33.6	29.2
RMS. deviations						
Bond lengths (Å)	0.006	0.005	0.005	0.005	0.006	0.005
Bond angles (°)	1.199	0.776	0.807	0.775	0.793	0.792
Ramachandran favored (%)	96	96	96	96	97	96
Ramachandran outlier (%)	0	0	0	0	0	0
PDB ID	6LVQ	6L1S	6LOU	6LMY	6LOT	7C8S

References

1. Zhang, J.; Bajari, R.; Andric, D.; Gerthoffert, F.; Lepsa, A.; Nahal-Bose, H.; Stein, L.D.; Ferretti, V. The International Cancer Genome Consortium Data Portal. *Nat Biotechnol* **2019**, *37*, 367-369, doi:10.1038/s41587-019-0055-9.
2. Tate, J.G.; Bamford, S.; Jubb, H.C.; Sondka, Z.; Beare, D.M.; Bindal, N.; Boutselakis, H.; Cole, C.G.; Creatore, C.; Dawson, E., et al. COSMIC: the Catalogue Of Somatic Mutations In Cancer. *Nucleic Acids Res* **2019**, *47*, D941-D947, doi:10.1093/nar/gky1015.

© 2020 by the authors. Licensee MDPI, Basel, Switzerland. This article is an open access article distributed under the terms and conditions of the Creative Commons Attribution (CC BY) license (http://creativecommons.org/licenses/by/4.0/).
